# Secondary damage management of acute traumatic spinal cord injury in low and middle-income countries: A survey on a global scale (Part III)

**DOI:** 10.1016/j.bas.2022.101694

**Published:** 2022-12-05

**Authors:** Nicolò Marchesini, Andrés M. Rubiano, Francesco Sala, Andreas K. Demetriades, Oscar L. Alves

**Affiliations:** a Department of Neurosurgery, University Hospital Borgo Trento, Verona, Italy; b Neuroscience Institute, Universidad El Bosque, Bogotá, Colombia; c Meditech Foundation, Cali, Colombia; d Department of Neurosurgery, Royal Infirmary, Edinburgh, UK; e Department of Neurosurgery, Leiden University, the Netherlands; f Department of Neurosurgery, Centro Hospitalar de Gaia/Espinho, Vila Nova de Gaia, Portugal; g Department of Neurosurgery, Hospital Lusiadas Porto, Porto, Portugal

## Abstract

• In LMICs, several factor may affect the applicability of guidelines for secondary damage control of spinal cord injury.
• In LMICs, the use of steroids for spinal cord injury is heterogeneous and admissions to an intensive care units are limited.
• The delays for surgical decompression of spinal cord injury can be significan and vary across income and geographic region.
• Transfer times seem to be the most common reason for surgical delay in all income and geographic regions.
• Costs for surgery for spinal trauma may be a significant barrier to guideline adherence, especially in low-resource settings.

## Abbreviations

**EA&P**: East Asia and Pacific
**E&CA**: Europe and Central Asia
**HDU**: high-dependency unit
**HICs**: high-income countries
**ICU**: intensive care unit
**LA&C**: Latin America and the Caribbean
**LICs**: low-income countries
**LMICs**: low- and middle-income countries
**L-MICs**: lower-middle-income countries
**ME&NA**: Middle East and North Africa
**SA**: South Asia
**SSA**: Sub-Saharan Africa
**TSCI**: traumatic spinal cord injury
**TSI**: traumatic spinal injury
**U-MICs**: upper-middle income countries
**WFNS**: World Federation of Neurosurgical Societies

## 1 Introduction

Traumatic spinal injury (TSI) is a pathological condition that occurs when a sudden force is applied and the resulting energy is transmitted to the spinal elements, causing fractures and/or dislocations that in turn may acutely damage the spinal cord (traumatic spinal cord injury ​= ​TSCI). From a functional perspective, these events can produce motor, sensory and autonomic impairments which are the consequence of acute, sub-acute and chronic structural and biochemical insults. Primary injury occurs in the initial stage of TSCI and is associated with the direct destruction of neuronal, glial and axonal structures. Although clinical manifestations may suggest a complete disruption of neural networks, some connections may still remain intact during this initial phase (Anjum et al., 2020; Fan et al., 2018; Venkatesh et al., 2019; Eli et al., 2021; Okada et al., 2018). Prevention is the only way to avoid primary injury (Bellon et al., 2013; von Groote et al., 2014).

Secondary injury is triggered by primary injury and involves multiple biochemical mechanisms that last for several weeks after trauma and that can aggravate the functional and clinical condition or, with an optimistic perspective, halt improvements (Orr and Gensel, 2018; Ahuja et al., 2017; Sandrow-Feinberg and Houlé, 2015).

Medical therapies, cardiopulmonary management, surgical decompression and stabilization and rehabilitation are all measures that synergistically can be adopted to mitigate the impact of secondary damage on patients' outcomes and for this reason, most guidelines focus their recommendations on such topics (Hurlbert et al., 2013; Gelb et al., 2013; Fehlings et al., 2017a, 2017b; Zileli et al., 2020; Sánchez et al., 2020; Takami et al., 2020; Ryken et al., 2013; Peev et al., 2020). However, disparities across the different regions of the world related to distribution of resources, infrastructures, equipment, personnel and knowledge of the guidelines could be responsible for differences in the application of guidelines themselves (Mukhopadhyay et al., 2019; Calderón and Servén, 2014; WHO. World Health Statistics Organization, 2021). As a consequence of these inequities in the management and treatment of TSI and TSCI, patient's outcomes may be negatively affected. Recognition of these differences is the first step to propose alternatives or solutions in order to optimize the care of patients suffering from these conditions, with a global perspective.

## 2 Methods

An electronic survey of 34 questions was designed and distributed to physicians treating spinal trauma in LMICs (Appendix I). Google Modules (Google©) and Wenjuan© were the used platforms, as previously done in several online surveys (ainShaikh et al., 2021; Palanisamy and Battacharjee, 2019; Marchesini et al., 2022; Jiang et al., 2021). Dissemination strategy included emails, social media (Facebook, Instagram, Twitter, WhatsApp) and webinar presentations and respondents could access to the same questionnaire either by a common web link or a QR code. All the included questionnaires were filled out completely and incomplete modules were automatically excluded from analysis. Only partial aspects of the whole survey are dealt with and presented here. The questions examined in this article focused on some relevant phases of the in-hospital treatment of spinal trauma: the administration of high-dose steroids, the admission to Intensive Care Unit (ICU), the timing for surgery after trauma in cases of spinal cord injury, the causes of surgical delays, the costs of the operation and, finally, the equipment available both for surgery and conservative treatment and rehabilitation (questions from 21 to 29, Appendix I).

Data were obtained from 1154 respondents originating from 79 LMICs and results are presented stratified by country income (LICs ​= ​low-income, L-MICs ​= ​lower-middle-income, U-MICs ​= ​upper-middle income) as well as by geographical area (EA&P ​= ​East Asia and Pacific, E&CA=Europe and Central Asia, LA&C=Latin America and the Caribbean, ME&NA ​= ​Middle East and North Africa, SA=South Asia and SSA=Sub-Saharan Africa) according to the last Word Bank Classification. (World Bank)

Data were prospectively collected and the results were tabulated in a Microsoft Excel spreadsheet and descriptive statistics was performed by the same software.

## 3 Results

Of the 1154 answers received, most came from L-MICs (48.4%, 558/1154) and the most represented geographic area was LA&C (26%, 300/1154). Most respondents were male (90.2%, 1041/1154) and the most represented age group was 30–49 years (71.8%, 828/1154). The majority were specialists in Neurosurgery (48.9%, 564/1154) with an experience in managing spinal trauma longer than ten years (39.3%, 454/1154) (see Table 1).

**Table 1:** Main demographic information of the 1154 respondents to the questionnaire. (L-MICs ​= ​lower-middle-income countries, U-MICs ​= ​upper-middle-income countries, EA&P ​= ​East Asia and Pacific, E&CA=Europe and Central Asia, LA&C=Latin America and Caribbean, ME&NA ​= ​Middle East and North Africa, SA=South Asia, SSA=Sub-Saharan Africa).

Demographic	Total (%)
**Total (%)**	1154 (100)
**Sex**	
Male	1041 (90.2)
Female	113 (9.8)
**Age (years)**	
<25	3 (0.3)
25-29	67 (5.8)
30-49	828 (71.8)
50-69	246(21.3)
≥70	10 (0.9)
**Current job title**	
Consultant in Neurosurgery	564 (48.9)
Consultant in Orthopedics	361 (31.3)
Neurosurgery trainee	130 (11.3)
Orthopedic trainee	37 (3.2)
Other	62 (5.4)
**Experience with spinal trauma (years)**	
<5	393 (34.1)
5-10	307 (26.6)
>10	454 (39.3)
**Level of resources of the Institution**	
Low level	127 (11)
Medium level	594 (51.5)
High level	433 (37.5)
**Population served**	
<1 million	381 (33)
1–5 million	454 (39.3)
>5 million	319 (27.6)
**Spinal cord injury cases treatment**	
Yes, regularly	764 (66.2)
Yes, occasionally	375 (32.5)
No, never	15 (1.3)
**Income area**	
LIC	51 (4.4)
L-MIC	558 (48.4)
U-MIC	545 (47.2)
**Geographic area**	
EA&P	297 (25.7)
E&CA	98 (8.5)
LA&C	300 (26)
ME&NA	108 (9.4)
SA	223 (19.3)
SSA	128 (11.1)

### 3.1 Acute non-surgical management

The use of high-dose steroids in cases of TSCI was declared by 75% (865) respondents of the whole sample (always ​= ​14.3%; occasionally ​= ​60.7%), while only 25% declared to never use them. The use of steroids was less frequent among LICs respondents (never ​= ​52.9%) when compared with L-MICs (never ​= ​18.5%) and U-MICs (never ​= ​27.7%). The region with the highest rate of “always” answers was E&CA (27; 27.6%), whereas the highest rate of “occasionally” answers was from EA&P (223; 75.1%) and the highest rate of “never” was from SSA (60; 46.9%).

**Fig. 1:**
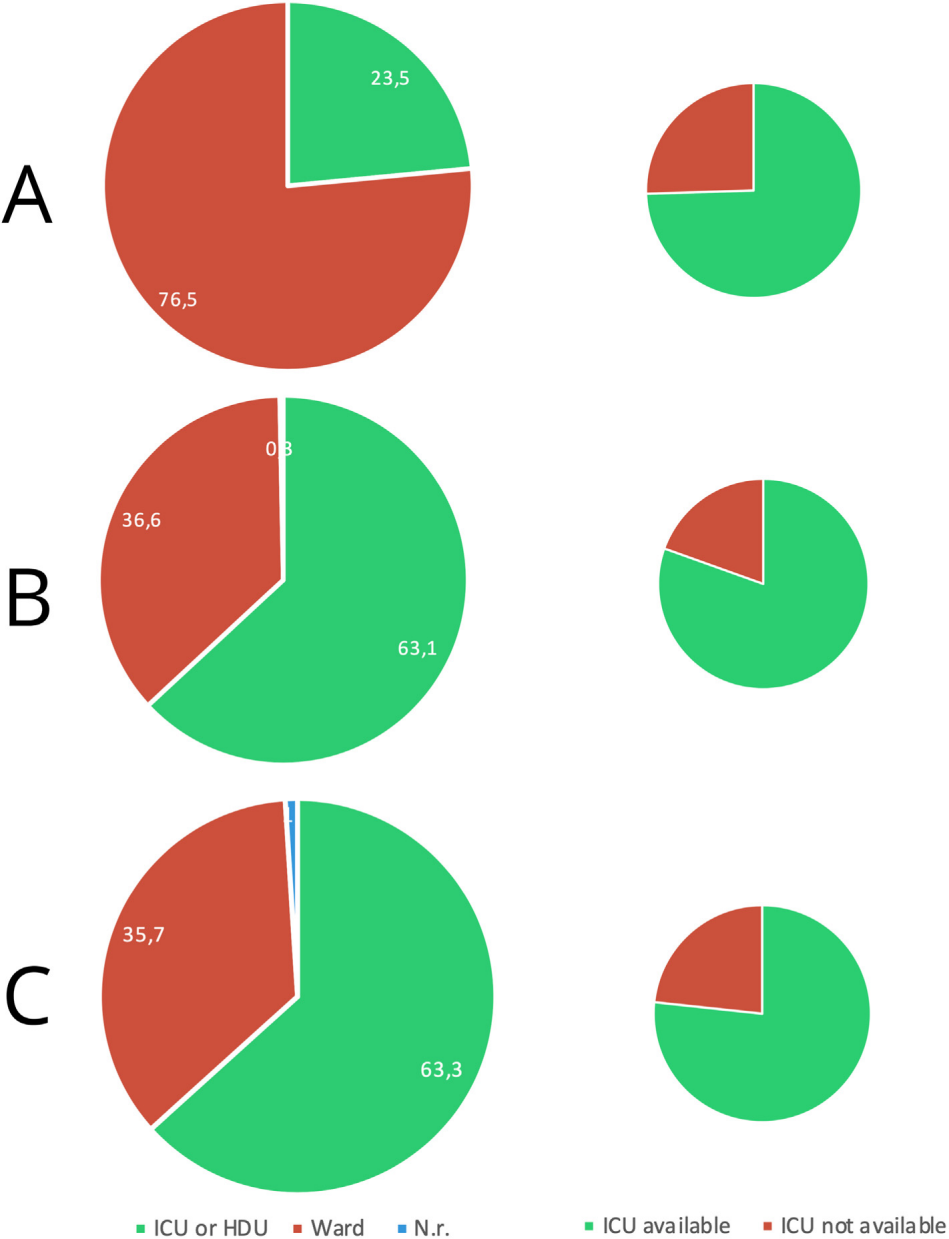
Rate of stated ICU and HDU/sub-intensive vs hospital ward admissions in TSCI cases (operated or treated conservatively) in LICs (A), L-MICs (B) and U-MICs (C), first column. Declared availability of an ICU facility in each income region, second column. N.r. ​= ​not responding or not receiving patients with spinal cord injury.

Overall, 61.4% (709) declared to treat TSCI patients in the acute phase in either an Intensive Care Unit or High-dependency/sub-intensive Unit while 38% (439) in a hospital ward (general or neurosurgical ward). The highest proportion of non-ICU and non-HDU/sub-intensive treatment was found in LICs (39; 76.5%) (see Fig. 1). According to the geographic area, the rate of non-ICU and non-HDU/sub-intensive was 50.8% (65) in SSA, 46.3% (139) in LA&C, 35.4% (105) in EA&P, 35.2% (38) in ME&NA, 31.4% (70) in SA and 22.4% (22) in E&CA.

### 3.2 Acute surgical management

Skull traction for closed reduction of cervical luxation was not used by 20.3% (234) of respondents in the entire sample (LICS ​= ​37.3%, L-MICs ​= ​13.4% and U-MICs ​= ​25.7%). The region with the least use was EA&CA (34.7% of no use at all).

**Fig. 2:**
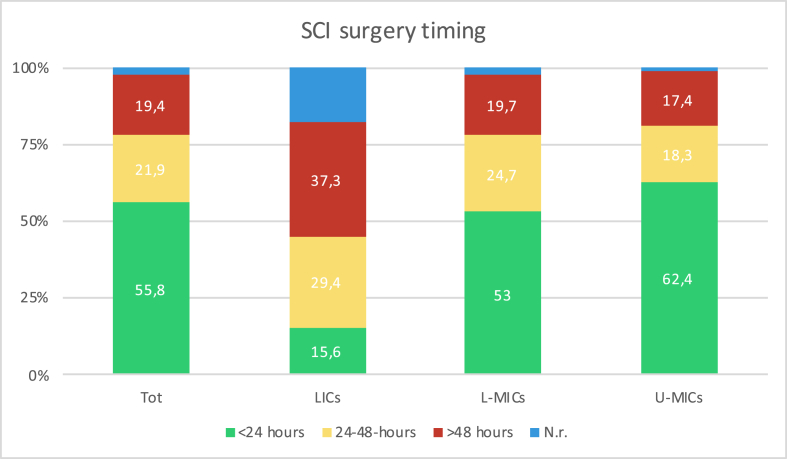
Timing for surgery in cases of spinal cord injury as declared by the 1154 respondents. Results are presented as for the whole sample (first column) and stratified according to the income region (second, third and last column). N.r. ​= ​not responding or not receiving spinal cord injuries.

**Fig. 3:**
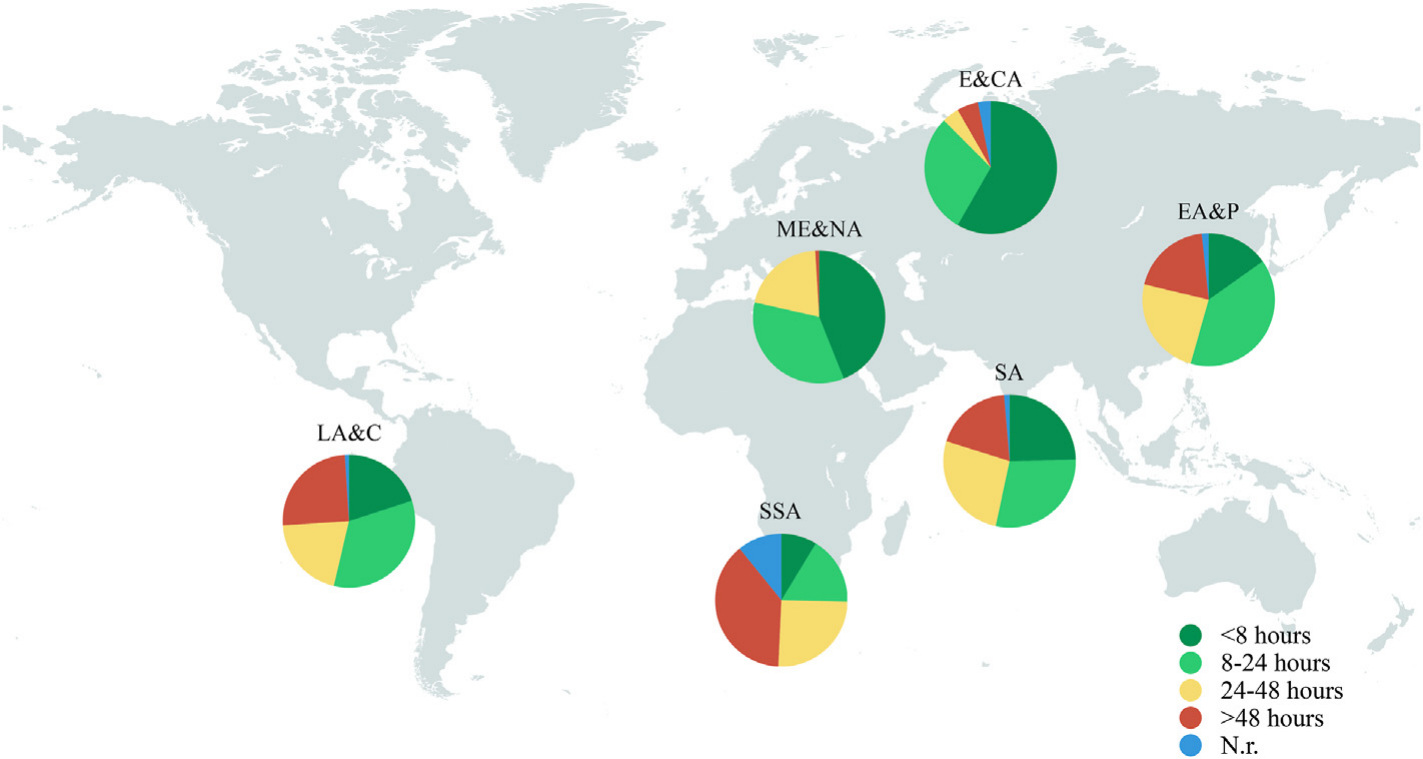
Regional differences in timing for surgery in spinal cord injury cases, as declared by the 1154 respondents. EA&P ​= ​East Asia and Pacific, E&CA=Europe and Central Asia, LA&C=Latin America and the Caribbean, ME&NA ​= ​Middle East and North Africa, SA=South Asia and SSA=Sub-Saharan Africa.

Overall, 55.8% (644) confirmed to be able to operate on spinal cord injury cases within 24 ​h from injury, however with a considerable difference in distribution amongst income and geographic regions (see Fig. 2, Fig. 3). In LICs, only 15.6% operate on these patients within 24 ​h while 29.4% do so between 24 and 48 ​h and 37.3% after 48 ​h from injury. The geographical area with the longer surgical delays was SSA (27.3% operated between 24 and 48 ​h and 33.6% after 48 ​h) while in E&CA 85.7% (84) stated to operate on TSCIs within 24 ​h. A high rate of surgical delays (>24 ​h) was also found in SA (45.3%), LA&C (45.3%), EA&P (43.7%) and ME&NA (21.3%).

Transfer times was the most commonly declared reason for surgical delays in the whole sample (709; 61.4%), followed by surgical equipment (451; 39.1%) and spinal imaging availability (315; 27.3%). Other reasons included operation room availability (7.9%), financial issues (4.3%) and clinical reasons (3.1%). Transfer times was the most frequently cited reason in all the income groups (LICs ​= ​66.7%; L-MICs ​= ​68.1%; U-MICs ​= ​54.1%) and geographical areas (EA&P ​= ​65.3%; E&CA ​= ​72.4%; ME&NA ​= ​63%; SA ​= ​71.3%; SSA ​= ​67.2%) except for LA&C where the commonest reason was surgical equipment availability (54%).

**Fig. 4:**
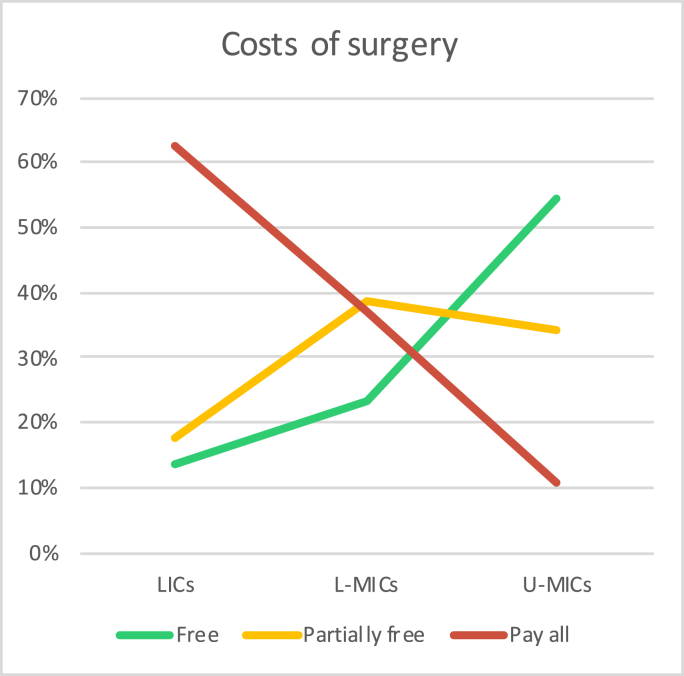
Proportion of answers regarding the costs of surgery for patients and their families as stated by the 1154 respondents. Results are stratified according by income region.

Overall, 37.5% (433) stated that surgery is completely free of charges for the patient, whereas 35.6% (411) recognized that patients have to partially contribute to the expenses and in 25.7% of cases (297) the costs of the operation were completely covered by the patients or their families. Considerable differences were found when stratifying the results according to the income and geographic group, with areas with lower income presenting a higher rate of paid surgery (see Fig. 4). SSA (85; 66%) and SA (97; 43.5%) were the regions where more patients have to pay all the surgery costs. Surgery was stated to be entire to be paid in 22% of cases in EA&P, 11.1% in ME&NA, 10.3% LA&C and 6.1% in E&CA.

Regarding surgical instrumentation, a complete availability of the listed equipment (see Table 2) was reported by 29.2% of respondents (337), 0% of LICs and 10.2% of SSA respondents. The instruments with greater differences in availability among income and geographic regions were C0-cervical instrumentation, C1–C2 instrumentation, anterior odontoid screw, thoracolumbar minimally invasive instrumentation, thoracolumbar anterolateral instrumentation, kyphotic-vertebroplasty.

**Table 2:** Results of the 1154 respondents to the questions relating the available equipment or facility for the surgical treatment of spinal trauma. ∗ ​= ​more than one option was available for the answer.

Surgical equipment										
Variable	Total (%)	LICs	L-MICs	U-MICs	EA&P	E&CA	LA&C	ME&NA	SA	SSA
**Total (%)**	1154(100)	51 (4.4)	558(48.4)	545(47.2)	297(25.7)	98(8.5)	300(26)	108(9.4)	223(19.3)	128(11.1)
**Equipment/facility∗**										
Surgical loops	529(45.8)	20(39.2)	254(45.5)	255(46.8)	152(51.2)	51(52)	149(49.7)	38(35.2)	92(41.3)	47(36.7)
Surgical microscope	736(63.8)	11(21.6)	366(65.6)	359(65.9)	220(74.1)	73(74.5)	186(62)	74(68.5)	145(65)	38(29.7)
Bipolar and electrocautery	976(84.6)	46(90.2)	481(86.2)	449(82.4)	246(82.8)	83(84.7)	255(85)	90(83.3)	193(86.5)	109(85.2)
Fluoroscopy	850(73.7)	31(60.8)	406(72.8)	413(75.8)	202(68)	70(71.4)	248(82.7)	83(76.9)	167(74.9)	80(62.5)
Anterior cervical instrumentation	864(74.9)	34(66.7)	437(78.3)	393(72.1)	230(77.4)	74(75.5)	213(71)	78(72.2)	175(78.5)	94(73.4)
C0-cervical instrumentation	616(53.4)	5(9.8)	313(56.1)	298(54.7)	155(52.2)	58(59.2)	166(55.3)	57(52.8)	140(62.8)	40(31.3)
C1–C2 instrumentation	566(49)	7(13.7)	275(49.3)	284(52.1)	139(46.8)	54(55.1)	145(48.3)	56(51.9)	135(60.5)	37(28.9)
Anterior odontoid instrumentation	441(38.2)	8(15.7)	202(36.2)	231(42.4)	105(35.4)	42(42.9)	109(36.3)	45(41.7)	111(49.8)	29(22.7)
Thoraco-lumbar posterior (open)	850(73.7)	37(72.5)	429(76.9)	384(70.5)	219(73.7)	72(73.5)	209(69.7)	84(77.8)	175(78.5)	91(71.1)
Thoraco-lumbar posterior (MIS)	439(38)	4(7.8)	218(39.1)	217(39.8)	132(44.4)	48(49)	98(32.7)	52(48.1)	94(42.2)	15(11.7)
Thoraco-lumbar anterior/lateral	471(40.8)	7(13.7)	220(39.4)	244(44.8)	113(38)	50(51)	124(41.3)	51(47.2)	101(45.3)	32(25)
Kypho/vertebroplasty	506(43.8)	2(3.9)	240(43)	264(48.4)	140(47.1)	62(63.3)	130(43.3)	66(61.1)	94(42.2)	14(10.9)
Intensive Care Unit	905(78.4)	38(74.5)	449(80.5)	418(76.7)	243(81.8)	77(78.6)	232(77.3)	81(75)	175(78.5)	97(75.8)
All of the above	337(29.2)	0(0)	153(27.4)	184(33.8)	95(32)	29(29.6)	85(28.3)	34(31.5)	81(36.3)	13(10.2)
None of the above	9(0.8)	1(2)	5(0.9)	3(0.6)	1(0.3)	0(0)	3(1)	0(0)	2(0.9)	3(2.3)

### 3.3 Conservative management and post-operative care

**Fig. 5:**
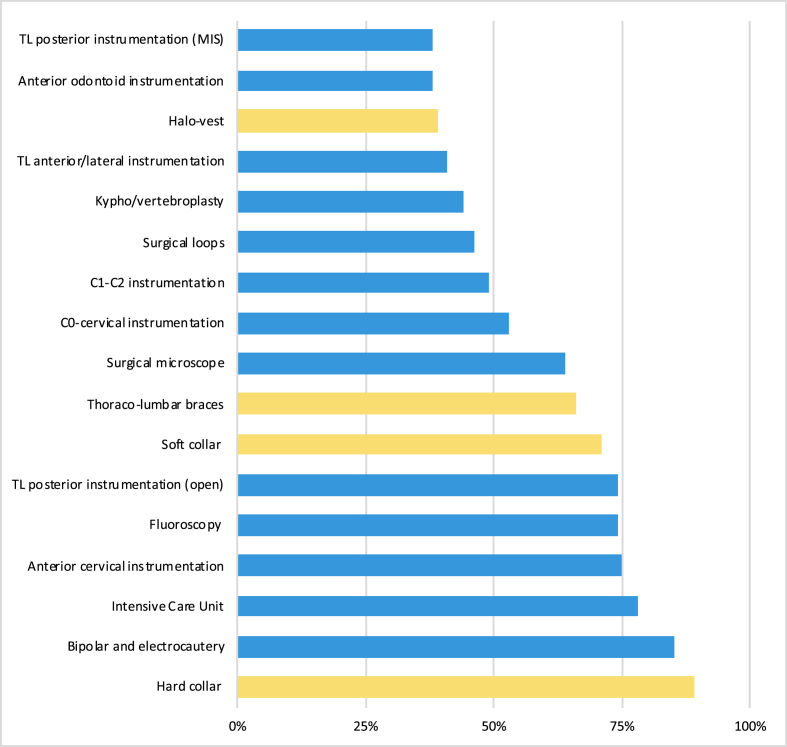
Availability of instruments and equipment for the management of spinal trauma as reported by the 1154 respondents. In blue, instrument for surgical treatment; in yellow, equipment for conservative management. TL ​= ​thoraco-lumbar; MIS ​= ​minimally invasive. (For interpretation of the references to colour in this figure legend, the reader is referred to the Web version of this article.)

The most widely available equipment for conservative treatment was hard cervical collar (89.3%), ranging from 82% (LA&C) to 94% (SA). Halo-vest could be used by 39.1% respondents (LICs ​= ​9.8%; L-MICs ​= ​38%; U-MICs ​= ​42.9%) (see Fig. 5).

**Fig. 6:**
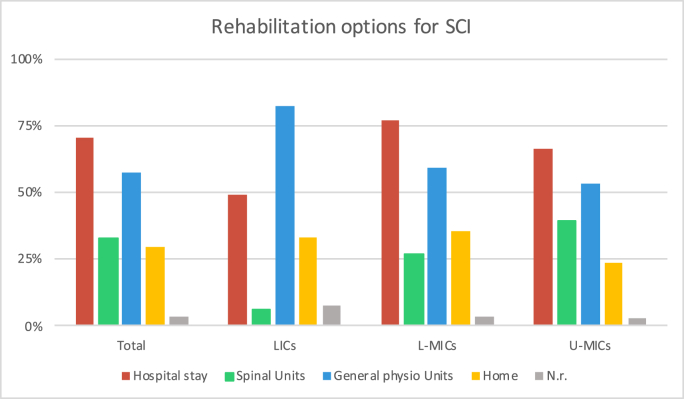
Type of available rehabilitation options for patients with spinal trauma as reported by the 1154 respondents. Results are presented as for the whole sample (first group of columns) and stratified according to the economic macro-area (second, third and last groups of columns). N.r. ​= ​not responding or not receiving spinal cord injuries.

Overall, the most commonly mentioned facility for rehabilitation was during the hospital stay (814; 70.5%), while a minority dispose of home rehabilitation (342; 29.6%) or dedicated Spinal Units (377; 32.7%). Considerable differences were found according to the income or geographic region (see Fig. 6). In LICs most reported to access to General physio Units (42; 82.4%) while only 5.9% (3) to dedicated Spinal Units. Overall, the availability of Spinal Units varied between 42.9% (E&CA) and 23.4% (SSA) while in-hospital rehabilitation was available for 56.5% (ME&NA) to 85.9% (EA&P) respondents. The rate of respondents stating to treat TSCIs in General physio Units ranged from 38.8% (E&CA) to 68.8% (SSA) while home rehabilitation did not exceed 39.8% (ME&NA)

## 4 Discussion

In general, noteworthy differences were found across income and geographic areas in the adherence to the recommendations focused on secondary injury management after TSI and TSCI in LMICs. The differences/ambiguities amongst guidelines could explain some of these discrepancies, but for the most part these are probably due to local dynamics.

Despite the promising effects of many new molecules in animal models of TSI, most failed to translate into positive clinical results in mitigating secondary damage in TSCI (Karsy and Hawryluk, 2019; Liu et al., 2021). In this arena, one of the most discussed therapies is the administration of high doses of methylprednisolone sodium succinate, an anti-inflammatory corticosteroid with putative neuroprotective effect (Sámano and Nistri, 2019). Its role as a therapeutic option for TSCI is supported by experimental animal studies, but clinical trials resulted in scarce evidence for a positive impact on motor outcomes regardless of the dose regimens used (Bracken et al., 1984, 1990, 1997; Canseco et al., 2021). Additionally, concerns have been raised due to the increased risk of complications associated with its use, like infections and pulmonary diseases (Bracken et al., 1984, 1990, 1997; John Hurlbert, 2000). As a result, guidelines are heterogeneous about this subject. The WFNS Spine Committee recommendations and the AOSpine guidelines accept the administration of high-dose methylprednisolone in selected young patients within 8 ​h from injury (Fehlings et al., 2017b; Takami et al., 2020). Conversely, other entities don't recommend its use at all (Hurlbert et al., 2013; Spinal, 2016; Roquilly et al., 2020). As the guidelines are not completely aligned, it may be inappropriate to discuss on the compliance with the guidelines regarding the use of steroids in LMICs. The relatively high rate of steroid use among responders is probably influenced by the partial recommendation of WFNS and AOSpine. Additionally, the differences across geographic and income regions that we found could be the reflection of such ambiguity rather than a matter of resources. However, as LICs and SSA were respectively the income and geographic areas with the least use, an economic role can't be a-priori excluded.

Instead, guidelines appear more uniform when recommending intensive cardiopulmonary management of TSCI, when indicated. Amongst others, this includes the use of devices to monitor cardiovascular and respiratory functions, the management of hypotension and bradycardia associated with spinal shock, and the maintenance of an optimized mean arterial blood pressure to secure spinal cord perfusion (Sánchez et al., 2020; Ryken et al., 2013; Roquilly et al., 2020; Hogg et al., 2021). Although dedicated Acute Spinal Cord Injury Centers have been shown to decrease overall mortality and the number and severity of complications, a general ICU setting is recommended as it provides adequate medical care after TSCI (Parent et al., 2011; Schwartzbauer and Stein, 2016). Accordingly, nearly 80% of our sample stated to have access in their Institution to an ICU, with rates ranging from 74.5% (LICs) to 80.5% (L-MICs) when considering the income area and from 75% (ME&NA) to 81.8% (EA&P) when examining the geographic area (see Fig. 1). Despite the widespread availability of ICU it is surprising that nearly 40% of the responders in the whole sample recognized to treat TSCIs in general or neurosurgical hospital wards, with rates reaching 76% in LICs. It's possible that in some countries other acute life-threatening diseases are prioritized in disfavor of TSCI due to shortage of ICU beds and limited financial resources. Possibly, patient triage is done according to the baseline functional status, the severity of disease and the chance of favourable outcome before ICU admission (Ribeiro et al., 2019). Although difficult to generalize in different contexts, data show that in LMICs, trauma patients are almost four times less likely to be admitted to an ICU, probably due to the complex management and high fatality rates of this condition (Blanch et al., 2016). Thus, it can be concluded that in many LMICs regions a significant proportion of TSCI patients are be sub-optimally treated, with clinical results that may be inferior to what could be achieved with an intensive acute medical care. This is confirmed by previous studies about other conditions which showed lower mortality rates in contexts where less restrictive criteria for ICU admissions apply (Blanch et al., 2016). ICU admission discrepancies between private and public facilities were also found (Ribeiro et al., 2019).

Surgery for acute TSCI is one of the cornerstone topics. The expression “time is spine” summarizes well the body of evidence that demonstrates the importance of early decompression and stabilization (if needed), to limit the effects of secondary damage. Early surgery increases the chances of neurological recovery and allows early mobilization for rehabilitation therapy (Badhiwala et al., 2018, 2019; Fehlings et al., 2012). Some recent studies shorter even further the limit of 24 ​h from injury for surgical decompression to 12 or 8 ​h with encouraging results, though data come from small series of patients (Burke et al., 2019; Jug et al., 2015). The current guidelines invariably recommend early decompression, whether by closed reduction or/and surgery, within 24 ​h (Fehlings et al., 2017a; Zileli et al., 2020; Spinal, 2016; Schleicher et al., 2018). The recent WFNS Spine Committee recommendations state that surgery within 8 ​h should be performed in most TSCI cases (Sánchez et al., 2020).

Although our sample is too small to generalize, according to our results one could assume that in LICs (where around 650 million people live) only one out of six TSCI patients is operated on within 24 ​h; in L-MICs (where approximately 3.3 billion people live) one out of two operations for TSCI is delayed for more than 24 ​h. Additionally, physicians from the overwhelming majority of geographic areas wouldn't be able, except in E&CA, to follow what is recommended by the guidelines about the surgical timing in a significant proportion of cases. We were able to show that in all income and nearly all geographic regions transport seems to be the most common reason for surgical delays. Although in our study we didn't discriminate direct from indirect transfers (from the scene or from the referring institutions), both could contribute to delays in the presentation to final care facility. It has been demonstrated that for general trauma patients direct transfers are associated with shorter delays and lower mortality and there is no reason to doubt that these considerations can be valid also for TSCIs (Boschini et al., 2016; Yohann et al., 2022). The development of trauma systems and protocols, including prehospital and primary care hospitals, may constitute valid strategies to narrow the gap between HICs and LMICs regarding surgery timing (Yohann et al., 2022). Additionally, the high cost of surgeries, supported entirely by patients in poor areas of the globe, constitute a barrier to perform surgery in due time. We recommend public policies that implement universal health care coverage (Kruk et al., 2018). Actually, the results of recent cost-effective studies suggest that surgery-related costs may be offset by a reduction in disability, and LMICs governments should be encouraged to conduct further spine trauma management and treatment cost-effectiveness analyses (Lessing et al., 2022).

Some responders declared general clinical conditions as a cause of surgical delay for TSCI. Even if in some cases severe polytrauma patients require stabilization before an operation, high injury severity score and polytrauma status shouldn't be considered per se as contraindications for early surgery in TSCI cases (Costa et al., 2021).

Despite all the efforts to reduce primary and secondary damage, TSCI is associated in a considerable proportion of cases with a broad spectrum of disabilities. Rehabilitation options aim to reduce complications and the impact on the daily life of such impairments. For these reasons, some guidelines include in their recommendations a section on rehabilitation issues besides more specific guidelines (Peev et al., 2020; Martin Ginis et al., 2018). Early transfer to specialized spinal units is in general advisable (Maharaj et al., 2016). However, according to our results, only for a minority of TSCI cases are referred to specialized rehabilitation centers, with comsiderable differences from region to region while general physiotherapeutic units seem more widely accessible.

As a magic bullet strategy that mitigates the effects of secondary damage has not yet been discovered, a multidisciplinary and preventive approach should be followed to improve the outcomes and the quality of life of patients who sustain a TSCI. Current guidelines resume the available best medical evidence and provide important tools to optimize the care offered to these patients. However, some of the recommendations devised to manage secondary damage across different phases of care seem to be unequally followed/followable worldwide. In the future, we consider it advisable to find, develop and adopt strategies to overcome the limits of real-life implementation of the existing guidelines for TSI and TSCI in LMICs, easing their contextualization and adoption.

## 5 Limitations

Several limitations may affect the current study, and this includes those that are common to this methodology. The survey was distributed using different means, including social media, emails and presentation at webinars. For this reason it was not possible to calculate response rate. The questionnaire was distributed only in English, reason why non-English spoken physicians could have chosen not to participate. As participation was optional, many of the responders may have a special interest in the topic when compared to non-responders, which introduces a selection bias. Our sample may be not exactly representative of all LMICs idiosyncrasies and scenarios, although our results include an unusually high number of respondents. Additionally, there is the risk of clustering of results with multiple responders from the same institution.

## 6 Conclusions

The adherence to guidelines for the management of secondary damage of TSCI worldwide appears to be far from homogeneous: most physicians treating TSCI in LMICs consider the use of high-dose steroids, although in most cases only occasionally; although ICUs are reported as available by most LMICs respondents, a considerable proportion still manage TSCI in hospital wards, with low-resource areas proclaiming the lowest ICU admission rate; a high volume of respondents appear to not operate on TSCIs within the desirable 24 or even 48 ​h time-windows, and delays in some areas seem to be considerable, with excessive transfer times being the most commonly acknowledged reason for surgical delays; rehabilitation options significantly vary across income and geographic areas, with dedicated spinal units being less available in low-resource settings. After the local reality is described, strategies should be designed to overcome the barriers of real-life implementation of the existing guidelines for TSI and TSCI in LMICs. This, in order to improve patients outcome and economical burdens.

## Declaration of competing interest

The authors declare that they have no known competing financial interests or personal relationships that could have appeared to influence the work reported in this paper.
